# SCUBE-1 as a Biomarker Predictor for the Home Follow-Up and Hospitalization of SARS-CoV-2 Patients

**DOI:** 10.3390/jcm15020637

**Published:** 2026-01-13

**Authors:** Selçuk Eren Çanakçi, Kenan Ahmet Turkdogan, Mustafa Kerem Ozyavuz, Faruk Celik, Mehmet Mesut Sonmez, Ibrahim Yilmaz, Ali Osman Arslan, Abdullah Emre Güner, Şakir Ümit Zeybek

**Affiliations:** 1 Department of Emergency Medicine, Alaaddin Keykubat University, 07425 Alanya, Turkey; selcuk.eren.canakci@gmail.com (S.E.Ç.); drturkdogan@gmail.com (K.A.T.); 2 Department of Molecular Medicine, Aziz Sancar Institute of Experimental Medicine, Istanbul University, 34452 Istanbul, Turkey; mkeremo@gmail.com; 3 Department of Medical Genetics, Alaaddin Keykubat University, 07425 Alanya, Turkey; celikfaruk@yahoo.com; 4 Molecular Medicine Research Center, Alanya Alaaddin Keykubat University, 07425 Alanya, Turkey; 5 Department of Orthopedics and Traumatology, Istanbul Haseki Training and Research Hospital, University of Health Sciences, 34734 Istanbul, Turkey; 6 Department of Biochemistry, Haseki Training and Research Hospital, Health Sciences University, 34096 Istanbul, Turkey; d.ibrahimyilmaz@hotmail.com; 7 Department of Medical Biology, Bolu Abant Izzet Baysal University Medical Faculty, 14280 Bolu, Turkey; aliosmanarslan@ibu.edu.tr; 8 Department of Public Health, Hamidiye Faculty of Medicine, University of Health Sciences, 34734 Istanbul, Turkey; abdullahemre.guner@sbu.edu.tr

**Keywords:** emergency department triage, COVID-19 severity assessment, neutrophil-to-lymphocyte ratio (NLR), inflammatory biomarkers, clinical outcomes

## Abstract

**Background/Objectives**: Severe Acute Respiratory Syndrome Coronavirus 2 (SARS-CoV-2) continues to pose a significant global health challenge due to its high transmissibility and potential for severe clinical outcomes. Early identification of patients at risk of hospitalization is essential for effective triage in emergency departments and for the optimal allocation of healthcare resources. **Methods:** This prospective study included 84 patients aged over 18 years who presented to the emergency department on 23 December 2020, with suspected SARS-CoV-2 infection. Initially, 100 patients were evaluated, and 16 were excluded based on predefined exclusion criteria. The mean age of the participants was 53.65 ± 13.62 years, and 39 (46.4%) were women. **Results:** At admission, the mean signal peptide, CUB domain, EGF (SCUBE-1) level among SARS-CoV-2 patients was 0.16 ± 0.08 ng/mL. There was no significant difference in SCUBE-1 levels between patient and control groups (n = 59 vs. 25), but levels differed significantly between hospitalized and home-treated patients (n = 37 vs. 22; *p* = 0.001). Neutrophil count (*p* = 0.001) and NLR (*p* = 0.010) were higher in patients than controls and also higher in hospitalized than home-treated patients (*p* = 0.003 and *p* = 0.015). ROC analysis revealed that SCUBE-1 predicted hospitalization with 84.6% sensitivity and 88.9% specificity. A positive correlation was observed between SCUBE-1 levels and length of hospital stay (*p* = 0.007, r = 0.554), with a median stay of 9.0 (5.0–11.0) days. **Conclusions:** SCUBE-1 levels were significantly associated with disease severity in SARS-CoV-2 patients and may serve as a promising biomarker to support clinical decision-making for hospitalization versus home-based management.

## 1. Introduction

Coronavirus Disease 2019 (COVID-19), caused by Severe Acute Respiratory Syndrome Coronavirus 2 (SARS-CoV-2), has exerted an extraordinary burden on global healthcare systems since its emergence in late 2019 [1]. Although vaccination strategies and therapeutic interventions have substantially reduced mortality, marked heterogeneity in clinical presentation persists, ranging from asymptomatic infection to severe disease requiring hospitalization and intensive care. Early identification of patients at risk for clinical deterioration remains a critical challenge, particularly in emergency department settings, where rapid triage, early risk stratification, and optimal allocation of limited healthcare resources can directly affect patient outcomes and hospital workflow efficiency. Indeed, accurate prognostication at the point of first clinical contact in the emergency department has been emphasized as pivotal in mitigating overcrowding and guiding decisions such as inpatient admission, antiviral therapy initiation, and monitoring intensity [2].

Numerous studies have explored hematological and inflammatory biomarkers to predict disease severity and clinical outcomes in COVID-19. Parameters such as the neutrophil-to-lymphocyte ratio (NLR), C-reactive protein, and D-dimer levels have been associated with adverse outcomes and increased mortality across diverse populations [3]. However, these conventional markers often lack sufficient specificity to reliably distinguish patients who require hospitalization from those suitable for outpatient management, particularly at early presentation to emergency care, where laboratory turnaround times may be delayed and clinical decisions must often be made within minutes to hours. Thus, there is a clear need for novel biomarkers that not only correlate with severity but also reflect distinct pathophysiological processes inherent to severe COVID-19 [4,5].

Accumulating evidence indicates that endothelial dysfunction, platelet activation, and COVID-19-associated coagulopathy lie at the heart of the disease’s progression and organ injury. SARS-CoV-2 infects host cells by binding to the ACE2 receptor, which is expressed not only in respiratory epithelium but also on vascular endothelial cells, thereby enabling widespread endothelial involvement [6]. Viral entry and subsequent immune activation lead to a cascade of endothelial injury, loss of anticoagulant properties, upregulation of adhesion molecules, and a shift toward a prothrombotic milieu. These processes contribute to microvascular occlusion, thromboinflammation, and multi-organ dysfunction, phenomena that have been repeatedly documented in both clinical and mechanistic studies of COVID-19 pathogenesis [7].

Signal peptide, CUB domain, EGF-like protein 1 (SCUBE-1) is a cell-surface glycoprotein expressed predominantly in endothelial cells and platelets and is released upon platelet activation and vascular injury. Soluble SCUBE-1 has been characterized as a biomarker of platelet activation and endothelial perturbation and has been associated with thrombotic burden in various cardiovascular and ischemic conditions [8]. In the context of COVID-19, preliminary evidence suggests that SCUBE-1 levels are significantly elevated in infected patients compared to controls, correlate with disease severity, and are associated with thrombotic complications and in-hospital mortality [9,10].

Given the centrality of endothelial injury and thromboinflammation in severe SARS-CoV-2 infection, SCUBE-1 represents a biologically plausible and mechanistically anchored marker that may capture key aspects of COVID-19 pathophysiology beyond traditional inflammatory indices [11,12]. Its measurement at the time of emergency department presentation could potentially enhance early risk stratification, inform clinical decision-making regarding inpatient versus outpatient management, and identify patients at high risk for progression to critical illness. Nevertheless, data regarding its clinical utility remain comparatively limited, necessitating further investigation [13]. Therefore, the present study aimed to evaluate serum SCUBE-1 levels in patients presenting to the emergency department with confirmed SARS-CoV-2 infection and to investigate its potential value in predicting the need for hospitalization and disease severity.

## 2. Materials and Methods

### 2.1. Research Design and Study Population

This study was designed as a single-center, prospective observational conducted in the emergency department. Adult patients (≥18 years) presenting with suspected SARS-CoV-2 infection during the study period were consecutively enrolled. Patients were classified according to polymerase chain reaction (PCR) results and clinical follow-up strategy (home follow-up or hospitalization), which constituted the primary clinical endpoint.

One hundred patients presenting to the emergency department with suspected SARS-CoV-2 infection were initially evaluated. A total of 59 patients with positive polymerase chain reaction (PCR) test results comprised the SARS-CoV-2 group, while 25 PCR-negative patients served as the control group. Sixteen patients were excluded based on the predefined exclusion criteria. Those in the SARS-CoV-2 group were further divided into two subgroups: those who were hospitalized and those who were followed up at home. Among the 59 PCR-positive patients, 37 were managed with home follow-up, whereas 22 required hospitalizations. Patients who were discharged were followed up at home for 30 days to determine whether further hospitalization was required. Laboratory findings, PCR test results, and length of hospital stay were recorded.

This study was approved by the Local Ethics Committee Institutional Review Board of Bağcılar Training and Research Hospital (Approval Date: 21 December 2020; Approval No: 2020/432). Written informed consent was waived by the ethics committee in accordance with the approved study protocol. All procedures were conducted in accordance with the principles of the Declaration of Helsinki.

### 2.2. Inclusion Criteria and Exclusion Criteria

Patients aged ≥18 years who presented to the emergency department with suspected SARS-CoV-2 infection and underwent PCR testing were eligible for inclusion. Patients with a confirmed PCR-positive result constituted the SARS-CoV-2 group, while PCR-negative patients with similar clinical presentations served as the control group. All included patients had complete clinical, laboratory, and follow-up data available at the time of analysis.

Patients who are pregnant and those with pulmonary thromboembolism, peripheral artery embolism, deep venous thrombosis, transient ischemic attack and/or cerebrovascular disease, acute coronary syndrome and coronary artery disease, myocarditis or pericarditis, previous cardiac or vascular interventions, heart valve disease, endocarditis, and renal failure (serum creatinine ≥ 3) were excluded. Patients aged < 18 years and those with chronic liver disease, malignancy, hematological disease or a need for blood transfusion in the last 6 months, rheumatic disease, and those receiving immunosuppressive therapy were also excluded.

### 2.3. Laboratory Design

Complete blood count (CBC) was measured upon admission to the emergency department using an automated hematology analyzer (Sysmex D-60, Istanbul, Turkey) within 45–60 min of sample collection. Blood biochemistry was analyzed using an automated chemistry analyzer (Beckman Coulter AU-680, Fullerton, CA, USA) following standard laboratory protocols.

### 2.4. Plasma SCUBE-1 Test

Venous blood serum samples were collected in EDTA-containing tubes and centrifuged at 3000 rpm for 10 min to separate the plasma. The resulting plasma samples were aliquoted and stored at −30 °C until analysis. On the day of measurement, frozen samples were thawed at room temperature and analyzed for signal peptide, CUB domain, EGF-like domain-containing protein 1 (SCUBE-1) concentrations using a commercial enzyme-linked immunosorbent assay (ELISA) kit (Human SCUBE-1 ELISA Kit (Catalog No. E-EL-H5405, Lot No. KVYMF26CJ6; Elabscience, Wuhan, China), according to the manufacturer’s instructions. All samples were processed under identical conditions, and laboratory personnel were blinded to patients’ clinical data. The ELISA has a detection range and sensitivity suitable for quantitative determination of human SCUBE-1, with intra-assay and inter-assay coefficients of variation within the acceptable range, ensuring precision and reproducibility.

### 2.5. Statistical Analysis

Normality of continuous variables was assessed using the Shapiro–Wilk test. Pearson or Spearman correlation analyses were applied as appropriate based on data distribution.

Whether the continuous variables were normally distributed was determined by the Shapiro–Wilk test. Categorical variables are expressed as percentages. Variables with normal distribution are presented as standard deviation whereas those without normal distribution are presented as median (25–75%). The β value and its 95% confidence interval (CI) were also calculated. The discriminative ability of SCUBE-1 in determining patients who have SARS-CoV-2 at hospital was determined using the area under the receiver operating characteristic (ROC) curve. A *p*-value of <0.05 was considered statistically significant.

Due to the absence of prior data on SCUBE-1 levels in SARS-CoV-2 patients at the time of study design, a formal a priori sample size calculation could not be performed. The study population was therefore determined by the number of eligible patients presenting during the study period.

## 3. Results

The baseline characteristics of the control and SARS-CoV-2 patient groups are summarized in Table 1, and the characteristics of hospitalized versus home-treated SARS-CoV-2 patients are presented in Table 2.

SARS-CoV-2 patients vs. control group: No significant differences were observed between the control and SARS-CoV-2 patient groups in lymphocyte count, mean platelet volume (MPV), alanine aminotransferase (ALT), aspartate aminotransferase (AST), fibrinogen, D-dimer, C-reactive protein (CRP), SCUBE-1 levels, age, or sex (*p* > 0.05). However, the neutrophil/lymphocyte ratio (NLR), white blood cell (WBC) count, and neutrophil count were significantly higher in the SARS-CoV-2 group compared to the control group (*p* < 0.05).

**Figure 1:**
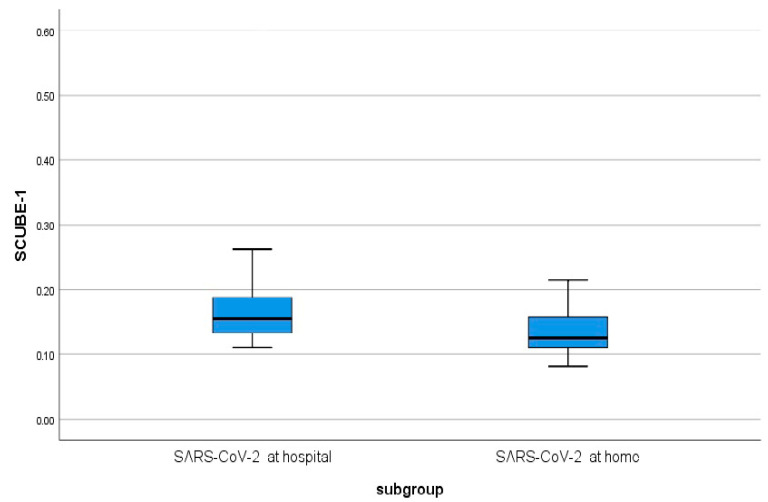
Distribution of SCUBE-1 among the COVID-19 patients.

Hospitalized vs. home-treated SARS-CoV-2 patients: Within the SARS-CoV-2 group, no significant differences were found between hospitalized patients and those followed up at home in WBC count, neutrophil count, MPV, ALT, AST, fibrinogen, D-dimer, CRP, age, or sex (*p* > 0.05). In contrast, NLR, lymphocyte count, and SCUBE-1 levels were significantly higher in hospitalized patients than in home-treated patients (*p* < 0.05). The distribution of SCUBE-1 levels according to treatment setting is illustrated in Figure 1.

**Figure 2:**
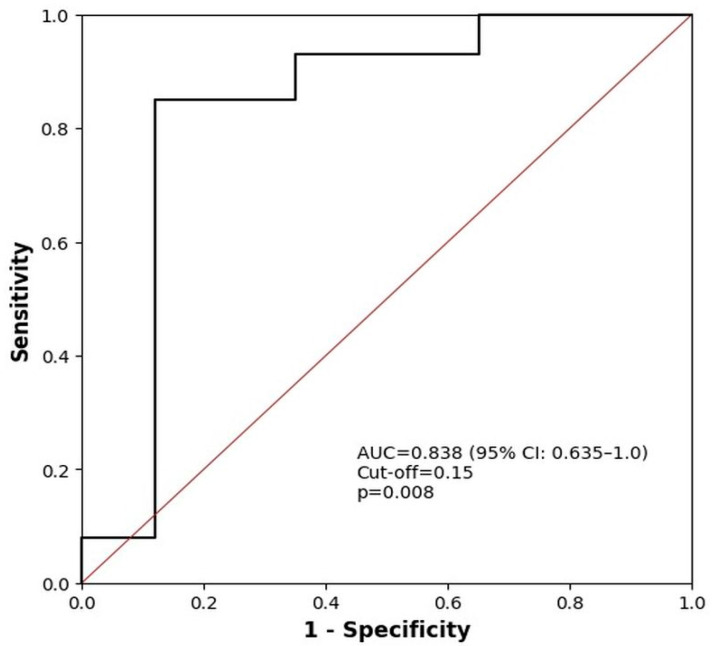
ROC analysis of SCUBE-1.

SCUBE-1 levels were significantly higher in hospitalized SARS-CoV-2 patients (0.14; range: 0.12–0.15) than in patients followed up at home (0.12; range: 0.11–0.14). Moreover, the specificity and sensitivity of SCUBE-1 were 88.9% and 84.6%, respectively. The ROC curve analysis of SCUBE-1 according to patient treatment is shown in Figure 2.

The average hospital stay was 9.0 (5.0–11.0) days, and a positive correlation was observed between the length of hospital stay and SCUBE-1 level (*p* = 0.007, r = 0.554). Within 15 days of follow-up, two patients treated at home required rehospitalization (on the third and sixth day)

## 4. Discussion

High patient volume, the proper organization of examination and treatment, and the identification of patients suitable for home follow-up are particularly important in terms of morbidity and mortality during a pandemic. To the best of our knowledge, no previous study has defined the primary role of SCUBE-1, an inflammatory biomarker, in patients with SARS-CoV-2. In this study, we demonstrated that SCUBE-1 levels may be associated with the clinical course of SARS-CoV-2 infection. Our findings revealed that SCUBE-1 levels were significantly higher in hospitalized patients compared to those managed at home. Furthermore, the positive correlation between SCUBE-1 and length of hospital stay suggests that this biomarker may have not only diagnostic but also prognostic value [14,15,16].

In our study, 73.8% of cases were mild to moderate and managed at home, whereas 26.2% were severe and required hospitalization. This distribution highlights the importance of clinical decision-making in managing patient load during a pandemic. In hospitalized patients, leukocyte and neutrophil counts were elevated, while lymphocyte counts were reduced; together with a marked increase in NLR, these findings underscore their role as strong indicators of disease severity [17,18].

Numerous studies have demonstrated the prognostic value of NLR in COVID-19. Kyala et al. reported that elevated NLR was associated with mortality [19]. Papanikolopoulou et al. showed that NLR and PLR could predict disease severity independently of immunosuppression status [20]. In our study, NLR was 2.7 in patients managed at home and in hospitalized patients, supporting its utility in guiding clinical decision-making.

Although the precise mechanisms underlying SCUBE-1 elevation in COVID-19 remain incompletely defined, growing evidence indicates that endothelial activation and platelet dysfunction are central features of COVID-19 pathophysiology, providing a biologically plausible link to our findings. Severe acute respiratory syndrome coronavirus-2 (SARS-CoV-2) induces endothelial injury and a pro-coagulant state characterized by microvascular thrombosis and increased platelet activation. SCUBE-1, a cell surface protein expressed in activated platelets and endothelial cells, is released during platelet activation and contributes to thrombus formation. Elevated SCUBE-1 levels in COVID-19 plausibly reflect exacerbated thrombo-inflammatory processes, supporting the clinical associations reported in this study [21,22,23,24].

Although our study did not include direct mechanistic experiments, accumulating evidence suggests that elevated SCUBE-1 reflects endothelial activation, platelet aggregation, and thrombo-inflammatory responses in COVID-19. SCUBE-1, expressed in platelets and endothelial cells, promotes platelet–endothelial adhesion and aggregation in thrombotic states [25], and elevated levels have been associated with disease severity, thrombotic complications, and in-hospital mortality in COVID-19 [9]. These observations provide a biologically plausible link between SCUBE-1 elevation and endothelial perturbation even in patients without overt cardiovascular disease [22,26], supporting the relevance of our clinical findings and highlighting the need for future mechanistic studies to clarify the molecular pathways involved

Recent studies support the diagnostic and prognostic role of SCUBE-1 in COVID-19 [27]. Reported significantly elevated SCUBE-1 levels in COVID-19 patients compared to controls, with a clear association with disease severity. Similarly, Özkan et al. compared SCUBE-1 and sCD40L levels and demonstrated that SCUBE-1 could serve as a distinctive marker in clinical diagnosis [28]. These findings are consistent with the results of our study. The considerations of our study include its single-center design and relatively small sample size. Nevertheless, our findings are in line with the literature demonstrating the diagnostic and prognostic value of SCUBE-1 in COVID-19. Larger, multicenter studies are needed to validate these results.

Although we excluded patients with known thromboembolic, inflammatory, and cardiovascular diseases, subclinical endothelial dysfunction or undiagnosed comorbidities may have influenced SCUBE-1 levels. Previous studies indicate that SCUBE-1 can reflect endothelial perturbations even in patients without overt cardiovascular disease [21,22,29]. This supports the biological plausibility that subtle endothelial perturbations may influence SCUBE-1 levels even in the absence of overt clinical disease. Additionally, medication use prior to or during hospitalization, which was not systematically collected in this study, may act as a residual confounder. Future studies incorporating detailed comorbidity and medication data are warranted to clarify these effects.

This study is not without some constraints. Although patients with major inflammatory, thrombotic, and rheumatic diseases were excluded, it was not feasible to systematically exclude all chronic inflammatory or autoimmune conditions in the emergency department setting during the COVID-19 pandemic. Therefore, the potential influence of unrecognized inflammatory conditions on SCUBE-1 levels cannot be entirely excluded and should be addressed in future studies with larger and more comprehensive patient group. Another important point to consider is that our study did not include critically ill patients requiring ICU admission or experiencing mortality. Consequently, SCUBE-1 could not be assessed as a predictor of severe or fatal outcomes. Nevertheless, our findings demonstrate its potential in distinguishing hospitalized from home-managed patients. Future studies with larger and more diverse cohorts, including critically ill cases, are warranted to clarify the prognostic role of SCUBE-1 in severe COVID-19.

## 5. Conclusions

In conclusion, SCUBE-1 levels were shown to be associated with disease severity and length of hospital stay in COVID-19 patients. When considered together with NLR, SCUBE-1 emerges as a novel biomarker that may aid clinical decision-making. The results of this study further suggest that SCUBE-1 is closely related to SARS-CoV-2 and may help fill the biomarker gap in both patients managed at home and those requiring hospitalization. It may even serve as a prognostic marker alongside NLR. Nevertheless, these findings should be validated by larger-scale, multicenter clinical studies to confirm the diagnostic and prognostic utility of SCUBE-1 in COVID-19.

## 6. Limitations

This study has several limitations. First, it was a single-center study with a relatively small sample size, and a formal a priori sample size calculation could not be performed due to the lack of previously published data on SCUBE-1 levels in SARS-CoV-2 patients at the time of study design. In addition, no patients experienced a fatal course or required intensive care; two patients were rehospitalized and subsequently followed up at home. Due to the early stage of the pandemic and the limited availability of detailed clinical data, correlations with comorbidities, other inflammatory markers, or vaccination status could not be assessed. We believe that more meaningful results may be obtained in future studies with larger cohorts and comprehensive clinical variables. This study was conducted at a single center with a relatively small sample size, which may limit the generalizability of the findings. Future multicenter studies with larger cohorts are warranted to validate these results

## Figures and Tables

**Table 1:** Baseline demographic characteristics and laboratory findings for the control and SARS-CoV-2 groups.

Baseline and Laboratory Variables for All Participants				
	All Participants n = 84	Control Group n = 25	SARS-CoV-2 Group n = 59	*p*-Value
Age, yr	55.9 ± 14.6	55.9 ± 14.6	54.4 ± 15.2	0.73
Sex, Female/Male	39/45	11/14	28/31	0.78
WBC, 10^3^/µL	7.8 (5.9–10.1)	10.6 (7.4–10.1)	7.2 (5.9–9.3)	<0.001
Neutrophil, 10^3^/µL	5.3 (3.3–8.2)	7.1 (5.3–4.9)	4.5 (3.3–8.2)	<0.001
Lymphocyte, 10^3^/µL	1.7 (1.3–2.1)	1.6 (01.3–3.2)	1.7 (1.4–2.1)	0.55
MPV, fL	10.2 (9.2–10.8)	10.0 (9.2–10.4)	10.3 (9.6–11.0)	0.09
NLR, %	3.5 (2.1–7.0)	4.2 (2.1–7.0)	2.8 (1.9–5.4)	0.01
ALT, mg/dL	23.3 (18–31)	23.5 (19.0–29.5)	23.0 (18.0–31.0)	0.65
AST, mg/dL	29.0 (20.5–31.5)	29.0 (20.5–31.5)	29.0 (21.0–31.5)	0.49
Fibrinogen, mg/dL	584 (473–649)	558 (486–644)	611 (473–649)	0.15
D-Dimer, ng/mL	885 (296–3140)	910 (330–3140)	860 (580–1415)	0.49
CRP, mg/dL	33.0 (14.8–123)	31.0 (14.8–57.5)	35.1 (26.7–123.0)	0.36
SCUBE1, ng/mL	0.15 (0.11–0.27)	0.17 (0.10–0.27)	0.13 (0.11–0.15)	0.31

yr: Year, WBC: White Blood Cell, MPV: Mean Platelet Volume, NLR: Neutrophil/Lymphocyte Ratio, ALT: Alanine Aminotransferase, AST: Aspartate Aminotransferase, CRP: C-reactive protein. Baseline demographic characteristics and laboratory findings for the control and SARS-CoV-2 groups. Continuous variables were compared using the independent samples *t*-test for normally distributed data or the Mann–Whitney U test for non-normally distributed data. Categorical variables were compared using the Chi-square or Fisher’s exact test as appropriate. The reported *p*-values correspond to these comparisons.

**Table 2:** Baseline demographic characteristics and laboratory findings for the SARS-CoV-2 group.

Baseline and Laboratory Variables for the SARS-CoV-2-Positive Patients			
	SARS-CoV-2 at Home n = 37	SARS-CoV-2 at Hospital n = 22	*p*-Value
Age, yr	56.1 ± 12.9	55.8 ± 15.3	0.45
Sex, Female/Male	18/19	9/13	0.93
WBC, 10^3^/µL	7.1 (6.1–8.0)	8.1 (5.7–12.3)	0.29
Neutrophil, 10^3^/µL	4.5 (3.8–6.9)	5.8 (2.5–9.9)	0.50
Lymphocyte, 10^3^/µL	2.0 (1.6–2.3)	1.2 (0.6–1.4)	<0.001
MPV, 10^3^/µL	10.1 (9.6–10.8)	10.3 (9.7–10.8)	0.92
NLR, %	2.7 (1.7–4.1)	7.4 (2.6–8.9)	0.02
UREA, mg/dL	35 (29–44)	36 (28–51)	0.43
ALT, mg/dL	25 (19–31)	21 (15–23.5)	0.76
AST, mg/dL	29 (23–31)	25 (20–35)	0.33
Fibrinogen, mg/dL	611 (481–653)	611 (476–667)	0.37
D-Dimer, ng/mL	940 (630–1270)	860 (296–1425)	0.51
CRP, mg/dL	33 (23–94)	53 (40–136)	0.22
SCUBE1, ng/mL	0.12 (0.11–0.14)	0.14 (0.12–0.15)	<0.001
PCR Volume, %	29.1 (24.3–30.8)	25.5 (24.1–30.1)	0.31

yr: Year, WBC: White Blood Cell, MPV: Mean Platelet Volume, NLR: Neutrophil-Lymphocyte Ratio, ALT: Alanine Aminotransferase, AST: Aspartate Aminotransferase, CRP: C-reactive protein, PCR: Polymerase Chain Reaction. Continuous variables were compared using the independent samples *t*-test for normally distributed data or the Mann–Whitney U test for non-normally distributed data. Categorical variables were compared using the Chi-square or Fisher’s exact test as appropriate. The reported *p*-values correspond to these comparisons.

## Data Availability

The data that support the findings of this study are available on request from the corresponding author.

## Abbreviations

The following abbreviations are used in this manuscript:

SARS-CoV-2	Severe Acute Respiratory Syndrome Coronavirus 2
NLR	Neutrophil/Lymphocyte Ratio
PCR	Polymerase Chain Reaction
SCUBE-1	Signal peptide-CUB-EGF-like protein 1
ELISA	Enzyme-Linked Immunosorbent Assay
MPV	Mean Platelet Volume
ALT	Alanine Aminotransferase
AST	Aspartate Aminotransferase
CRP	C-Reactive Protein
WBC	White Blood Cell
ROC	Receiver Operating Characteristic
